# Physics-informed NN-based adaptive backstepping terminal sliding mode control of buck converter for PEM electrolyzer

**DOI:** 10.1016/j.heliyon.2024.e29254

**Published:** 2024-04-04

**Authors:** Abdullah Baraean, Mahmoud Kassas, Md Shafiul Alam, Mohamed A. Abido

**Affiliations:** a Department of Electrical Engineering, College of Engineering and Physics, King Fahd University of Petroleum & Minerals, Dhahran 31261, Saudi Arabia; b Interdisplinary Research Center for Sustainable Energy Systems (IRC-SES), Research Institute, King Fahd University of Petroleum and Minerals, Dhahran 31261, Saudi Arabia; c Applied Research Center for Environment and Marine Studies, Research Institute, King Fahd University of Petroleum and Minerals, Dhahran 31261, Saudi Arabia; d SDAIA-KFUPM Joint Research Center for Artificial Intelligence (JRCAI), KFUPM, Saudi Arabia

**Keywords:** Adaptive backstepping terminal sliding mode controller, Buck power converter control, PEM electrolyzer, Physics-informed neural network (PINN)

## Abstract

This paper proposes an advanced control approach to controlling a DC-DC buck converter for a proton exchange membrane (PEM) electrolyzer within the framework of a direct current (DC) microgrid. The proposed adaptive backstepping terminal sliding mode control (ABTSMC) leverages a physics-informed neural network (PINN) to accurately estimate and compensate for system uncertainty. The composite controller achieves finite-time convergence of the tracking error by combining backstepping control and terminal sliding mode control (TSMC). The proposed PINN aims to optimize the unconstrained parameters by utilizing observed training points from the solution, ensuring the network accurately interpolates a limited portion of the solution. The efficacy of the proposed hybrid control method is validated using a hardware-in-the-loop (HIL) implementation under various test settings, ensuring the preservation of the actual performance of the PEM electrolyzer during testing. The experimental verification results demonstrate that the proposed control method exhibits greater benefits, such as a faster dynamic response and greater robustness against parameter uncertainties than improved sliding mode-based controllers. In situations where operational conditions change, a rapid response is achieved within a mere 0.025s of settling time, exhibiting a minimal percentage overshoot of about 17.5% and presenting minimal fluctuations.

## Nomenclature

ABTSMC: adaptive backstepping terminal sliding mode control
BSMC: backstepping sliding mode controller
DC: direct current
DHLRNN: double hidden layer recurrent neural network
HIL: hardware-in-the-loop
ISMC: improved sliding mode-based controller
MPC: model predictive control
MSE: mean-squared-error
NN-ATSMC: neural network and an adaptive terminal sliding mode controller
PEM: proton exchange membrane
PID: proportional-integral-derivative
PINN: physics-informed neural network
PWM: pulse width modulation
RESs: renewable energy sources
SMC: sliding mode controllers
TI: Texas Instrument
TSMC: terminal sliding mode control

## 1 Introduction

The utilization of renewable energy sources (RESs) is key to mitigating climate change and securing a sustainable future for our planet. Wind and solar power have evolved as viable alternatives to fossil fuels, offering environmentally friendly and plentiful energy sources. Nevertheless, these sources possess certain limitations, including intermittent and geographic restrictiveness. To overcome these challenges and effectively harness renewable energy, exploring energy storage options like hydrogen is crucial. Hydrogen, the most abundant element in the universe, is a remarkable solution with immense potential and can be integrated as an energy carrier to effectively store and transport renewable energy [1,2].

While hydrogen systems have showcased promise in various tests, larger-scale implementation is yet to be witnessed. One of the significant obstacles hindering their widespread adoption is the cost associated with electrolyzers. Electrolysis, especially through PEM electrolyzers, requires electricity to drive the electrolytic process. This technology has been evolving since the 1960s to overcome the difficulties faced by other electrolyzers [3,4].

When combining RESs with electrolysis, it is imperative to consider multiple design restrictions. These include intermittent operations due to the reliance on weather conditions for energy production, a high DC bus voltage reaching hundreds of volts, and the need for a reduced DC voltage for the PEM electrolyzer [5]. Therefore, a step-down DC-DC converter with a suitable controller is required [5,6]. While PEM electrolyzers are ideal for responding to dynamic operations and absorbing energy during transient states, a control algorithm that can handle large reference signal variations is necessary. With the rapid advancement of power electronics converters, they become commonplace in power systems [7]. The DC-DC buck converter has garnered significant attention due to its high efficiency and simple structure and has found widespread use in wind energy systems [8], DC microgrids [9], DC motor drives [10], photovoltaic systems [11], and energy storage systems [12].

The main goal of the controller for the buck converter is to produce a signal that adjusts the output voltage to match the desired value. However, the buck converter system is vulnerable to various uncertainties and disturbances, including changes in load [13], fluctuations in the reference value [14], and variations in input voltage [15]. Consequently, achieving precise regulation of the output voltage has become a difficult endeavor, leading researchers to concentrate on the development of advanced control methods.

Early studies indicate that linear control techniques, such as proportional-integral-derivative (PID) control with fixed frequency and frequency regulation [[16], [17], [18], [19]], is commonly employed in the design of buck converter controllers. Although PID control provides a simple framework and can achieve precise tracking in the absence of disturbances, it's essential to note that it displays linear behavior [20]. This linearity can pose challenges to system robustness. This is because the converters are typically modeled using a state-space averaging approach, resulting in a model that depends on the operating point and is predominantly valid within its proximity. Consequently, these controllers are unsuited for dynamic operating conditions involving significant signals. Recent research efforts have thus concentrated on the development of advanced nonlinear control strategies for buck converters, including sliding mode controllers (SMC) [21,22], finite-time state-feedback controllers [23], and robust controllers [24].

SMC, as a nonlinear control technique, is widely preferred in converter applications due to its effective handling of disturbances [25]. Researchers in Ref. [26], addressed the overall design challenge of SMC for voltage tracking control, while [27] introduced a double integral term in the sliding surface to minimize tracking errors. It should be noted that SMC composed of a linear combination of the system's state variables can only guarantee the asymptotic convergence of tracking errors [28]. To tackle this limitation, TSMC was developed, ensuring the finite-time convergence of tracking errors through the inclusion of a terminal function. In the context of a power converter application, TSMC was employed in Ref. [29] to achieve a faster transient response. Furthermore, a modified fast TSMC approach was devised in Refs. [[30], [31], [32]] to enhance the dynamic behavior of converter systems. Additionally [33], applied a neural network and an adaptive terminal sliding mode controller (NN-ATSMC) to a DC-DC boost converter, ensuring error convergence and minimal chattering effect. The main drawback of these controllers lies in the fact that, when the disturbance decreases, they often generate excessive control input. This can lead to undesired control chattering effects or even actuator saturation. To address this issue, the authors in Refs. [34,35] have developed a robust tracking controller based on barrier function adaptive sliding mode (BFASM) for linear motor (LM) positioners.

The backstepping control approach has proven effective for nonlinear systems in strict feedback form, simplifying control design through the recursive design of virtual control laws for each subsystem [36]. Researchers have explored multiple strategies using the backstepping technique to optimize control performance in converter systems. One approach is the utilization of a single-loop observer-based control, as suggested in Ref. [37]. Another strategy involves employing second-order sliding mode control in combination with backstepping, as presented in Ref. [38].

Several researchers have explored model predictive control (MPC), with some proposing innovative methods for directly regulating the output voltage of a boost converter while minimizing an objective function, as in Ref. [39] or controlling the switching states of a buck converter without additional modulation using finite control set MPC, as in Ref. [40]. However, conventional MPC struggles with rejecting disturbance and uncertainty, leading to significant estimation errors. To overcome this, adaptive control has been utilized to estimate model parameters as in Ref. [41], and an adaptive projection algorithm has been proposed to ensure the boundness of the estimated term in Ref. [42]. Unfortunately, these methods can be unreliable when dealing with fast-varying parameters or other parameter variations. A more practical solution to handle system uncertainty is the neural network control method, which utilizes the nonlinear mapping capability of the activation function in the hidden layer of the neural network to estimate complex nonlinear functions [43].

The extraordinary growth of data accessibility and computing resources has brought about remarkable advancements in neural network learning and data analysis. Despite these achievements, analyzing intricate physical or engineering systems can be prohibitively expensive, leading to inadequate data and challenges in making informed decisions with limited information. Even cutting-edge technologies, such as deep neural networks, lack robustness and cannot ensure convergence when working with limited data [44]. However, by incorporating physics principles into machine learning and using PINN, we unlock a potent approach with enormous potential to drive scientific breakthroughs and revolutionize leveraging data. A recent publication introduced a new framework for PINN in power system applications [45]. The framework simplifies neural network structures, achieving high accuracy with significantly less training data. It can determine dynamic states and uncertain parameters in a fraction of the computational time required by conventional methods.

To create an efficient and effective nonlinear controller for the converter used in this application, it is necessary to model the PEM electrolyzer and understand its dynamic behavior [46]. However, conducting experimental tests on a physical PEM electrolyzer can be challenging and lead to deterioration [47]. To overcome this limitation, we designed a PEM electrolyzer digital twin in this study to evaluate the converter's performance and control.

Based on extensive research, a cutting-edge ABTSMC-PINN controller has been proposed, developed, and implemented for a buck power converter that is linked to a PEM electrolyzer. The major contributions and key benefits of the proposed work can be summarized as follows.

• PINN-based strategy is proposed to predict the nonlinear functions of the proposed system. This not only handles model uncertainty but also significantly enhances tracking performance. The PINN incorporates the physical laws governing the system, which is less susceptible to overfitting, enabling it to generalize unseen data more effectively.
• ABTSMC is designed to achieve convergence in a finite time and simplify control structure. Incorporating a switching control term proves to be an effective strategy for offsetting external disturbances and compensating for network approximation errors. This integration results in heightened steady-state accuracy and improved performance in rejecting disturbances. These combined advancements work in harmony to improve the overall efficiency and effectiveness of the control system.

The structure of this work is as follows: In Section 2, we present the DC-DC buck converter coupled with a PEM electrolyzer model. Moving on to Section 3, we detail the design of an ABTSMC controller implemented with PINN and conduct a stability analysis. To assess the effectiveness of the proposed system, we carry out a hardware-in-the-loop implementation in Section 4. To conclude, in Section 5, we summarize and discuss the outcomes and implications of our study.

## 2 System modelling

**Fig. 1:**
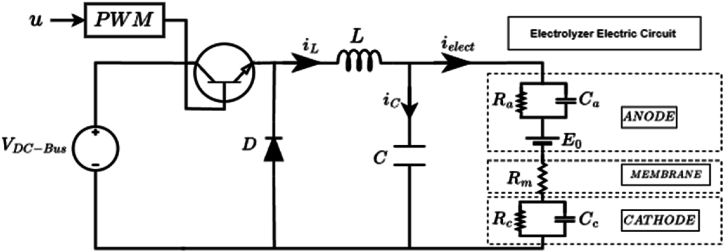
Proposed DC-DC buck converter connected with a PEM electrolyzer.

In this paper, the hydrogen production system is viewed as a component of a comprehensive DC microgrid. The system comprises a DC bus voltage, a proposed DC-DC buck converter, and a PEM electrolyzer, all illustrated in Fig. 1. The succeeding sections will provide a detailed breakdown of each component.

The existing literature on conversion systems for RES and PEM electrolyzers does not adequately address the specific characteristics and constraints of both. This research aims to fill this gap by proposing a novel approach that considers RES characteristics such as high input DC voltage (in case of solar PV and wind systems) with fast variations, as well as PEM electrolyzer constraints such as low DC voltage supply to prevent overshoots during transients. Additionally, the proposed system aims to achieve good efficiency conversion and robustness against faults. The novelty of this research lies in the design of an ABTSMC that can manage wide input voltage variations while imposing dynamic behavior since the converter response is independent of both the source and the load.

### 2.1 DC bus voltage dynamic model

Accurately modeling the DC bus voltage dynamics is vital for analyzing and controlling DC microgrid systems. A detailed description of the DC bus voltage dynamic model helps understand the system's behavior under different operating conditions.

Several factors affect the dynamics of the DC bus voltage. To accurately represent these dynamics, a mathematical framework is employed to model the voltage dynamics that takes into account RESs power injections, energy storage systems power outputs, and loads power demands. Voltage variations are also influenced by the system's inherent capacitance as well as the characteristics of the power electronic converters used in the microgrid.

(1) $$C_{DC-Bus}\frac{dV_{DC-Bus}}{dt}=\sum \left(P_{gen}-P_{load}\right)+I_{DC}\left(V_{DC-Bus}-V_{DC-Bus-ref}\right)$$

In this context, $C_{DC-Bus}$ denotes the aggregate capacitance of the DC bus, $\frac{dV_{DC-Bus}}{dt}$ signifies the temporal variation in the DC bus voltage, $I_{DC}$ represents the total DC bus current, and $V_{DC-Bus-ref}$ denotes the reference voltage for the DC bus. Net power injections from different sources are accounted for by $\sum \left(P_{gen}-P_{load}-P_{loss}\right)$.

This equation is important for understanding and evaluating the dynamic behavior of the DC bus voltage in a microgrid system. Allowing the development of strategies to maintain the DC bus voltage within desired levels.

### 2.2 Proposed buck DC-DC converter model

The mathematical representation of the buck converter shown in Fig. 1 can be derived by applying Kirchhoff's law and the state averaging method, as described in (2).

(2) $$\left\{ \begin{array}{c} \frac{di_{L}}{dt}=-\frac{1}{L}V_{elect}+\frac{V_{DC-Bus}}{L}u\\ \frac{dV_{elect}}{dt}=\frac{1}{C}i_{L}-\frac{1}{R_{elect}C}V_{elect} \end{array}\right.$$

The variables $V_{elect}$ and $i_{L}$ respectively denote the voltage of the electrolyzer and the current through the inductor L. u∈[0,1] is the control input, also known as duty cycle, which is compared against some triangular signal of fixed frequency to produce the pulse width modulation driving signal.

The dynamic model (2) can be expressed in terms of the state variables, which are the output voltage and its derivatives as shown in (3).

(3) $$\left\{ \begin{array}{c} {\dot{V}}_{elect}=\frac{1}{C}i_{L}-\frac{1}{X_{eq}C}V_{elect}\\ {\ddot{V}}_{elect}=-\frac{V_{elect}}{LC}-\frac{{\dot{V}}_{elect}}{R_{elect}C}+\frac{V_{DC-Bus}}{LC}u \end{array}\right.$$

Take $F_{1}=-\frac{V_{elect}}{LC}-\frac{{\dot{V}}_{elect}}{R_{elect}C}$ , and $F_{2}=\frac{V_{DC-Bus}}{LC}$ as the model uncertain terms, which will be estimated using the proposed PINN.

Obtaining accurate model parameters in a practical system is challenging due to variations in the environment and inaccuracies in measurements. These factors will result in system uncertainty, which will unavoidably degrade the voltage control accuracy of the buck converter. Thus, considering the aforementioned uncertainties, $F_{1}$ and $F_{2}$ are reformulated as:

(4) $$F_{1}=F_{1,0}+\Delta F_{1}$$

(5) $$F_{2}=F_{2,0}+\Delta F_{2}$$

The variables $F_{1,0}$ and $F_{2,0}$ represent the nominal components, whereas $\Delta F_{1}$ and $\Delta F_{2}$ represent the uncertain terms, which encompass variations in both parameters and disturbances. The uncertain terms exhibit robust nonlinearity, and their fluctuations are hard to predict. The proposed PINN is specifically developed to accurately estimate the nonlinear functions $F_{1}$ and $F_{2}$, in order to effectively address system uncertainty. Subsequently, the two estimated functions are incorporated into the framework of the proposed ABTSMC controller. Accordingly, the general model for the proposed system can be rewritten as:

(6) $$\left\{ \begin{array}{c} {\dot{V}}_{elect}=\frac{1}{C}i_{L}-\frac{1}{R_{elect}C}V_{elect}\\ {\ddot{V}}_{elect}=F_{1}+F_{2}u \end{array}\right.$$

### 2.3 PEM electrolyzer dynamic model

Utilizing the HIL technique with a PEM electrolyzer can serve as a valuable means of validating the functionality of the proposed hydrogen production system and its control algorithm prior to conducting physical experiments. This approach eliminates the risk of damage to an actual electrolyzer from overshoots or current and voltage ripples, as well as the need for hydrogen storage in the laboratory. To ensure the effectiveness of this method, the model must be capable of accurately replicating the actual behaviors of a PEM electrolyzer.

**Fig. 2:**
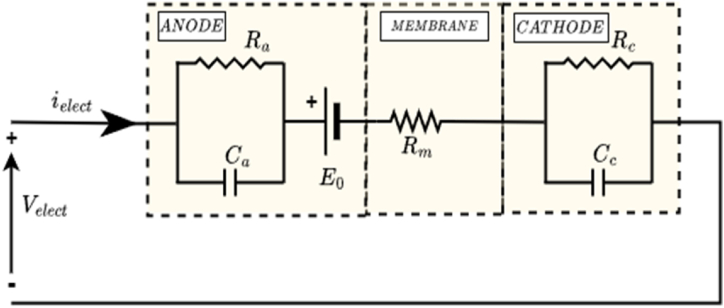
Equivalent Electrical circuit of a PEM electrolyzer.

Fig. 2 depicts the electrical circuit of the PEM electrolyzer, consisting of six components, including a DC voltage source, a resistor, and two RC branches. Table 1 outlines commercial PEM electrolyzer specifications and parameters. Two RC branches ($R_{a}$, $C_{a}$, and $R_{c}$, $C_{c}$) emulate the dynamic responses at the anode and cathode sides, as shown in Fig. 2. Additionally, the ohmic loss at the membrane is modeled by the resistor $R_{m}$, while the energy transformed into hydrogen (reversible voltage $E_{0}$) is represented by a DC voltage.

**Table 1:** PEM electrolyzer specifications & parameters [49].

Specifications	Value
Power (rated)	400W
Stack Voltage	8V
Stack Current (range)	(0−50)A
Number of Cells in the stack	3
Parameters	Value
Membrane Resistance $R_{m}$	0.09Ω
Anode Resistance $R_{a}$	0.333Ω
Cathode Resistance $R_{c}$	0.033Ω
Anode Capacitance $C_{a}$	33.33F
Cathode Capacitance $C_{c}$	33.30F

The suitability of a dynamic model for a PEM electrolyzer is extensively examined in Ref. [48]. The model presented in this study is constructed using an equivalent electrical circuit, as explained in Ref. [49]. Notably, the model is designed to mimic a real-life 400W PEM electrolyzer in both form and function. This means that the model accounts for the static and dynamic operation of the electrolyzer, as well as the electrochemical reactions that take place at the anode and cathode.

## 3 Proposed control system

To enhance the performance of the proposed PEM electrolyzer system and handle design uncertainties, a nonlinear adaptive controller has been developed. The proposed controller effectively regulates the system's response to voltage variations. Its primary function is to efficiently manage current absorption and prevent excessive voltage levels. By offering precise and resilience to dynamic changes, the controller greatly improves the reliability of the hydrogen production system even in the presence of external disturbances and variations in parameters. The adopted control technique is a modified backstepping SMC approach. It incorporates a terminal sliding surface and an adaptive law to accurately replicate the dynamic behavior of the system. As a result, the controller achieves enhanced control performance.

### 3.1 Backstepping sliding mode controller design

Backstepping control is based on breaking down a complex nonlinear system into smaller subsystems. This approach involves the iterative design of virtual control terms for each subsystem, which reduces the control design's complexity.

The symbol $V_{elect-ref}$ represents the reference voltage value. As per the backstepping control strategy, the variables $e_{1}$, $e_{2}$ are defined as the backstepping variables as following:

(7) $$\left\{ \begin{array}{c} e_{1}=V_{elect}-V_{elect-ref}\\ e_{2}={\dot{V}}_{elect}+K_{1}e_{1}-{\dot{V}}_{elect-ref} \end{array}\right.$$

Providing that $K_{1}>0$, $e_{2}$ represents the virtual control term. We consider the subsequent Lyapunov function:

(8) $$V_{1}=\frac{1}{2}{e_{1}}^{2}$$

Based on (7), (8), it can be inferred that:

(9) $${\dot{e}}_{1}=e_{2}-K_{1}e_{1}$$

Combining the derivative of $V_{1}$ with the dynamic model (3) produces:

(10) $${\dot{V}}_{1}=-\;K_{1}{e_{1}}^{2}+e_{1}e_{2}$$

If $e_{2}=0$, then ${\dot{V}}_{1}$ ≤ 0 is attained as a negative semi definite function. As a result, $e_{2}⟶0$ will be designed in the subsequent phases.

The defined Terminal sliding surface is characterized as follows [50]:

(11) $$s=e_{2}+K_{2}{{\dot{e}}_{2}}^{K}\;1<K>2$$

Where $K_{2}$ is a positive constant to enforce $e_{2}$ to converge to zero and K is also a positive constant between 1 and 2 which is important in the terminal sliding mode control to avoid the inherent singularity. These two control parameters should be chosen optimally to accelerate the convergence speed of $e_{2}$.

(12) $$\dot{s}=F_{1}+F_{2}u+K_{1}{\dot{e}}_{1}-{\ddot{V}}_{elect-ref}+K_{2}K{{\dot{e}}_{2}}^{\left(K-1\right)}{\ddot{e}}_{2}$$

Hence, the ideal control law u is formulated according to (13), effectively driving the system states towards the specified sliding surface from any initial position.

(13) $$u=\frac{1}{F_{2}}\left(-F_{1}-K_{1}{\dot{e}}_{1}+{\ddot{V}}_{elect-ref}-\int_{0}^{t}\left({\frac{1}{K_{2}K}{{\dot{e}}_{2}}^{\left(2-K\right)}+K}_{3}\left(s+K_{4}\text{sgn}\left(s\right)\right)\right)dt\right)$$

where $K_{3}$ and $K_{4}$ are constants with positive values.

As per the Lyapunov theorem, if all the model parameters are known for the dynamic model in (3), the sliding surface is selected as described in (11), and the control rule is designed as stated in (13), Consequently, the assurance of stability in the closed-loop system is established.

### 3.2 Adaptive backstepping terminal sliding mode controller using PINN

In practical applications, it can be difficult to obtain exact model parameters due to environmental fluctuations, leading to system uncertainty. To address this, a control law called the backstepping sliding mode controller (BSMC) will be designed. However, the BSMC controller can only guarantee asymptotic convergence to zero for voltage tracking errors. The controller design uses both the TSMC and the proposed PINN to adapt unknown functions ($F_{1}\;\&\;F_{2}$) in order to robustly account for system uncertainty and achieve tracking error's finite-time convergence.

**Fig. 3:**
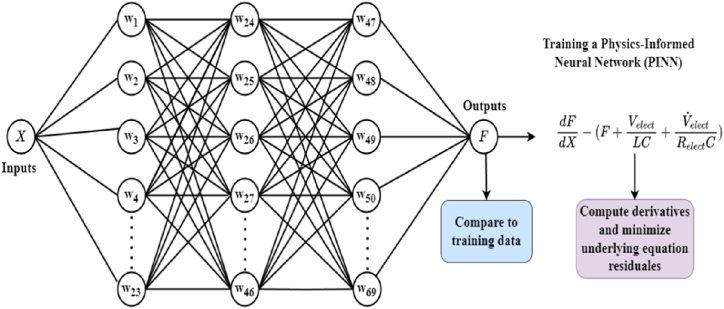
Physics-informed neural network (PINN) schematic.

In order to estimate the unknown functions, the objective is to optimize the network's unconstrained parameters, represented by the $w^{\prime }$ s in the proposed PINN diagram as in Fig. 3, to interpolate a limited portion of the solution. This is achieved by utilizing certain observed training points from the solution, thereby ensuring that the network's predictions are in close proximity to the available data. Typically, this task is accomplished by reducing the mean-squared-error (MSE) between the model's predictions ($F_{1-\text{PINN}}$) and the training data points.

(14) $$\min\frac{1}{N}\sum_{i}^{N}{\left(F_{1-PINN}\left(X_{i},W\right)-F_{1}\left(x_{i}\right)\right)}^{2}$$

Employing a solely data-driven approach may entail notable drawbacks. Although the standard neural network effectively captures the physical phenomenon within the range of the experimental data, it lacks the ability to extrapolate beyond the scope of the training data. It could be contended that a comprehensive understanding of the scientific problem cannot be achieved solely by depending on the data.

In scenarios where prior knowledge regarding the physics of the underlying problem exists, such as that described in Equation (3), researchers are exploring methods to integrate this scientific knowledge into machine learning frameworks, considering the limitations of conventional machine learning approaches. A potential approach for addressing our problem is to employ PINN. The proposed approach involves incorporating the fundamental differential equations into the loss function during the neural network training process, thereby enabling the accurate extrapolation of the complete solution beyond the confines of the provided training data points.

The process involves sampling a set of input training locations $\left(\left\{X_{j}\right\}\right)$ and feeding them through the network. The subsequent step involves the computation of the gradients of the neural network's output in relation to its input at these specific locations. This process is typically analytically feasible for the majority of neural networks and can be conveniently executed through auto differentiation. Finally, the residual of the fundamental differential equation is calculated utilizing the aforementioned gradients, and subsequently incorporated as an additional component within the loss function.

The validation test set assesses the ability of the neural network to predict based on new conditions that were not part of the training set. MSE or the $R^{2}$ value are common quantitative measures of the fit. Deep learning algorithms may be deployed across a wide variety of computing infrastructure or cloud-based services.

This amounts to using the following loss function to train the network:

(15) $$\min\frac{1}{N}\sum_{i}^{N}{\left(F_{1-PINN}\left(X_{i},W\right)-F_{1}\left(x_{i}\right)\right)}^{2}+\frac{1}{M}\sum_{j}^{M}{\left(\frac{dF_{1-PINN}\left(X_{j},W\right)}{dX}-\left(F_{1-PINN}\left(X_{j},W\right)+\frac{V_{elect}}{LC}+\frac{{\dot{V}}_{elect}}{R_{elect}C}\right)\right)}^{2}$$

Including the "physics loss" term within the loss function serves to reinforce the adherence of the network's predicted model to the fundamental laws of physics. This incorporation significantly boosts the reliability and credibility of the learned solution by ensuring it aligns more closely with established physical principles.

Achieving effective training and testing of the PINN for controlling the proposed PEM electrolyzer system relies heavily on the availability of a suitable training dataset. This dataset is curated through a comprehensive study and analysis of the system's characteristics under control, as detailed in Ref. [51]. The training process persists until the desired error (ε) is achieved, employing a learning rate (η) of 1e-3. After finishing the training, the PINN is ready to be deployed for various tasks. Fig. 3 visually depicts the network architecture and its components.

The training parameters of the Proposed PINN are presented in Table 2. The network undergoes offline training mode. PINN training involves the utilization of randomly generated data, ensuring comprehensive coverage across the entire search space of the estimated functions. The primary objective of PINN is to effectively establish the correlation between five inputs (L, C, $R_{elect\;}$, $V_{elect}$, and $V_{DC-Bus}$) and their respective functions ($F_{1}and\;F_{2}$). Subsequently, the two estimated functions are seamlessly integrated into the framework of the proposed ABTSMC controller.

**Table 2:** Training parameters of the proposed PINN.

Parameters	Value
Input Neurons	5
Hidden Layers	3
Hidden Neurons	23
Output Neurons	2
Iteration	1000
Method	LM-Based Back-Propagation (Modified)
ε	$10^{-07}$
η	$1^{-03}$

After the training phase, the PINN is subjected to testing utilizing different sets of input data. This critical phase aims to verify the network's capacity to accurately forecast the unknown functions within the PEM electrolyzer system. The efficiency of the PINN in capturing the system's behavior and creating reliable predictions is thoroughly tested by comparing its predictions to known values or experimental data.

### 3.3 Stability analysis

**Fig. 4:**
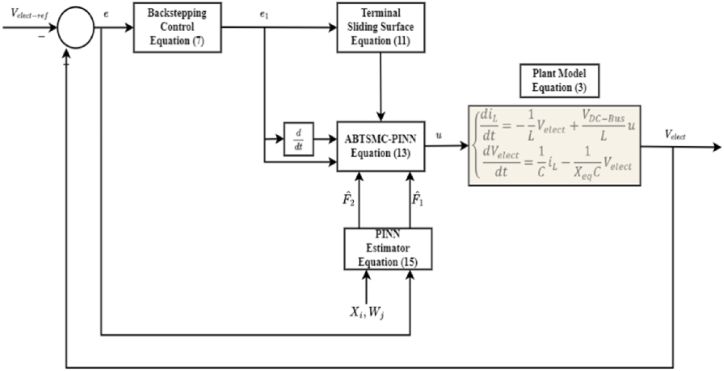
Proposed ABTSMC-PINN controller block diagram.

The block diagram in Fig. 4 shows the proposed approach structure, where the PINN is used to estimate the unknown functions ($F_{1}\;\&\;F_{2}$). In addition, the hyper-parameters of the PINN are trained offline utilizing adaptive principles based on the Lyapunov stability theorem. Thus, the system's inherent uncertainty can be effectively reduced and balanced by integrating the aforementioned estimates into the controller design.

To prove the asymptotic stability of the proposed controller, let us define a new Lyapunov function:

(16) $$V_{2}=\frac{1}{2}{e_{1}}^{2}+\frac{1}{2}s^{2}$$

By differentiating $V_{2}$ and then combining it with the designed control input in (13), we may obtain:

$${\dot{V}}_{2}=-K_{1}{e_{1}}^{2}+e_{1}e_{2}+s\left(K_{2}\left(e_{2}-K_{1}e_{1}\right)+F_{1}+F_{2}u+K_{1}{\dot{e}}_{1}-{\ddot{V}}_{elect-ref}\right)$$

$$=-K_{1}{e_{1}}^{2}+e_{1}e_{2}-{\gamma s}^{2}-\gamma \beta \left|s\right|$$

(17) $$=-e^{T}Qe-\gamma \beta \left|s\right|$$

Here ${e=\left[\begin{array}{cc} e_{1} & e_{2} \end{array}\right]}^{T}$ and $Q=\left[\begin{array}{cc} K_{1}+\gamma {K_{2}}^{2} & {\gamma K}_{2}-\frac{1}{2}\\ {\gamma K}_{2}-\frac{1}{2} & \gamma \end{array}\right]$.

The matrix Q is assured to be a positive-definite matrix by the careful selection of γ, $K_{1}$ and $K_{2}$ which will render the derivative of the defined Lyapunov function $V_{2}$ negative definite:

(18) $${\dot{V}}_{2}=-e^{T}Qe-\gamma \beta \left|s\right|\leq 0$$

By guaranteeing that $V_{2}$ is positive definite, we ensure that the voltage tracking error does not grow indefinitely. And by demonstrating that its time derivative ${\dot{V}}_{2}$ is negative definite or negative semi-definite, the error tends to decrease over time, ensuring asymptotic convergence to zero.

To prove the stability of the proposed controller as designed in Equation (13), the overall positive-definite Lyapunov function $V_{2}$ is considered. This allows for a thorough evaluation of the control stability. To guarantee the finite-time convergence of $V_{2}$ according to Theorem 1, it is verified that the derivative of $V_{2}$ is negative semi-definite as in Equation (18), with the condition that all $K_{i}$ are chosen positive.

Theorem 1 *If the Lyapunov-candidate-function*V*is globally positive definite*, *the equilibrium isolated and the time derivative of the Lyapunov-candidate-function is globally negative definite*:

(19) $$\dot{V}\left(x\right)<0\;\forall x\in R^{n}\backslash \left\{0\right\}\text{,}$$

*Then the equilibrium is proven to be globally asymptotically stable*.

*If these conditions are met*, *the stability of the voltage tracking error and the closed-loop system's stability can be ensured*.

## 4 HIL experimental implementation

To evaluate the performance and effectiveness of the proposed ABTSMC-PINN during transient operations, an experimental scenario was conducted. This scenario involved two dynamic operation conditions that represented significant variations in the DC bus voltage, allowing for the assessment of the system's response across the nonlinear characteristic of the electrolyzer. The specifications of the considered scenario for the proposed system are detailed in Table 3.

**Table 3:** System specifications.

Parameters	Value
Input DC bus voltage $V_{DC-Bus}$	100–200 V
Output electrolyzer voltage $V_{elect}$	8V
Buck converter Output capacitor C	4400μF
Buck converter Output inductor L	200μH
Switching Frequency $f_{s}$	40KHz

**Fig. 5:**
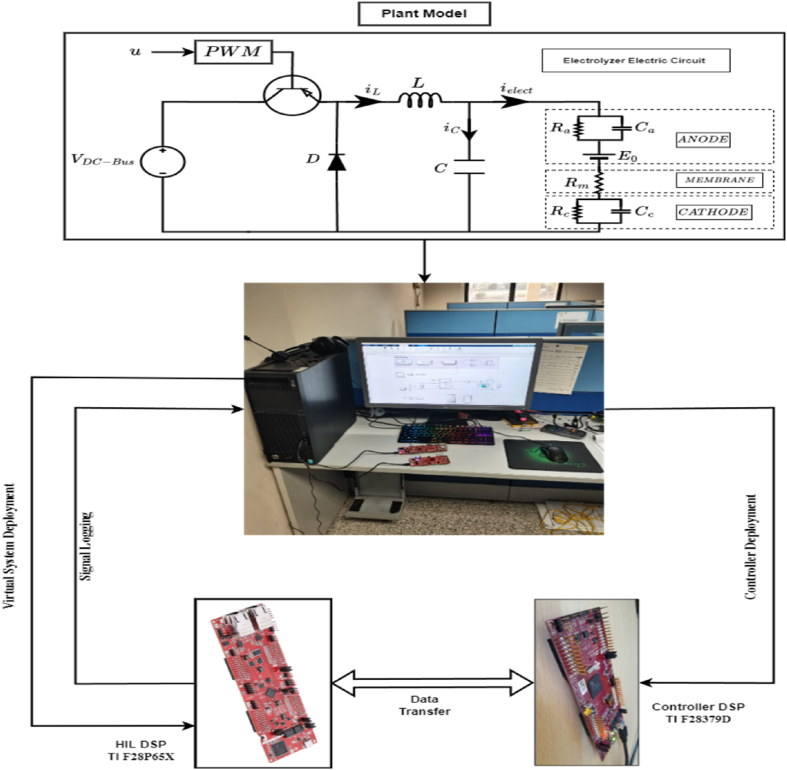
Proposed HIL Test pad.

As depicted in Fig. 5, the HIL setup is implemented using two DSPs from Texas Instrument (TI) . One of these DSPs functions as the control logic (C-HIL), while the other DSP embeds the proposed virtual system, serving as a HIL. The C-HIL DSP board acquires control signals (PWM) from an external device through GPIOs ports, which are used to drive the virtual system. Meanwhile, the HIL DSP provides feedback on the state variables of the system ($V_{elect},i_{elect}$) by utilizing HRPWM in conjunction with multiple analog low-pass filters to generate an equivalent analog signal.

Thanks to the advanced DSP technology utilized in the HIL system, updating the discrete system at a sampling rate of approximately 2.5μs (400KHz using a single DSP core) is achievable. As a result, it is possible to accurately replicate even the high-frequency behavior of the virtual buck converter with electrolyzer system being tested. This tool proves valuable in analyzing control logic behavior in challenging scenarios or during faulty conditions, and also serves as an effective means of testing control logic prior to implementation in real-world applications.

Additionally, the discrete model utilized in the HIL DSP can serve as a digital twin on the secondary core of the controller DSP. This enables monitoring of the parameters of the actual system and alerts in the event of any discrepancy between the behavior of the actual and virtual systems, paving the way for predictive maintenance.

To demonstrate the accuracy of the output waveform, which can be achieved using the proposed low-cost HIL setup, various results have been reported for the proposed system.

### 4.1 Electrolyzer Power Demand Variations

**Fig. 6:**
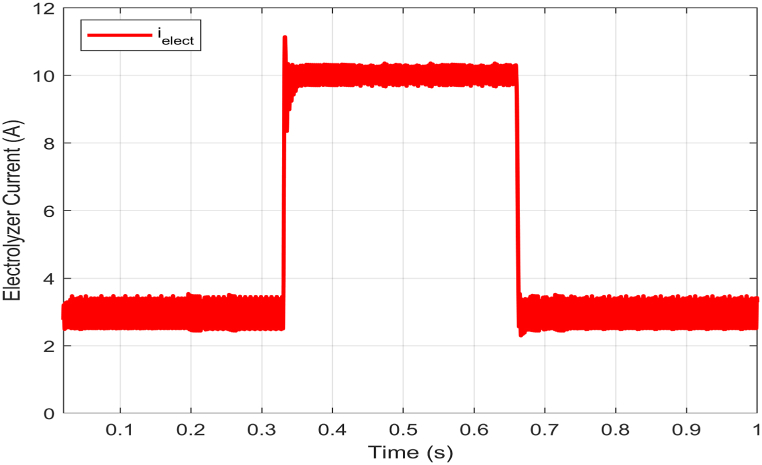
Electrolyzer current ($i_{elect}$) response with electrolyzer power demand variations.

The objective of the experiment is to maintain a constant voltage across the electrolyzer terminals while altering its current. This test serves to evaluate the robustness of the proposed controller in response to changes in the electrolyzer's power demand. The scenario involves adjusting the current reference of the PEM electrolyzer $i_{elect}$ between 3A and 10A as shown in Fig. 6 while maintaining a constant DC bus input voltage of 150V.

The experimental test conducted using the proposed HIL setup measured the responses of the electrolyzer voltage $V_{elect}$, and electrolyzer current $i_{elect}$. The figures below show the results obtained for this test.

**Fig. 7:**
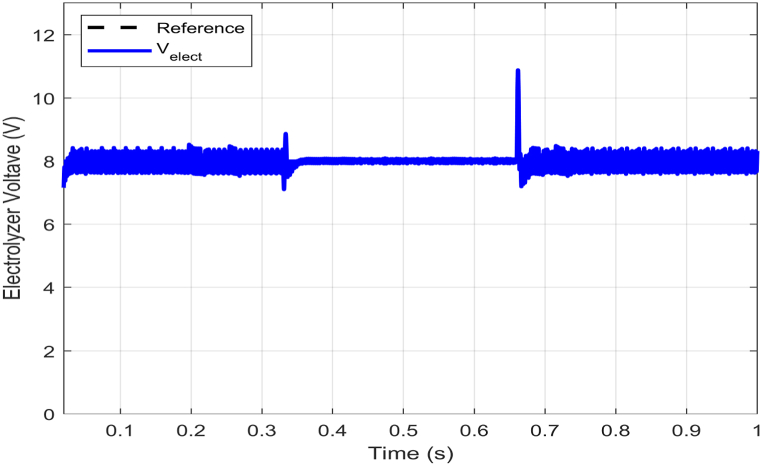
Electrolyzer voltage ($V_{elect}$) response with electrolyzer power demand variations.

It is worth noting that the measured electrolyzer voltage remains relatively stable at 8V with some marginally variations despite changes in current. Additionally, the proposed model for the PEM electrolyzer operates in normal voltage mode when $i_{elect}<7\;A$. However, when $i_{elect}$ exceeds 7A, the model switches to constant voltage mode. Therefore, this explains why there may be significant fluctuations in voltage response during normal modes, as seen in Fig. 7.

### 4.2 Electrolyzer Reference Voltage Variations

**Fig. 8:**
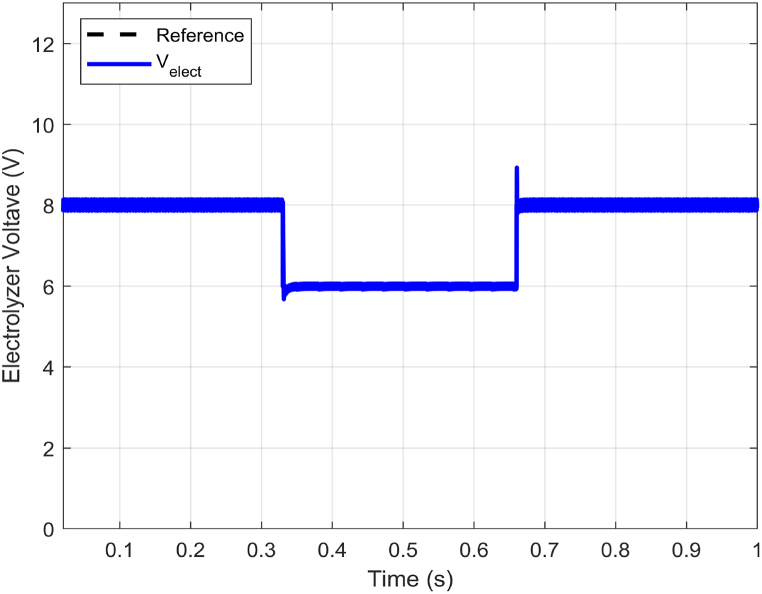
Electrolyzer voltage ($V_{elect}$) response with electrolyzer reference voltage variations.

**Fig. 9:**
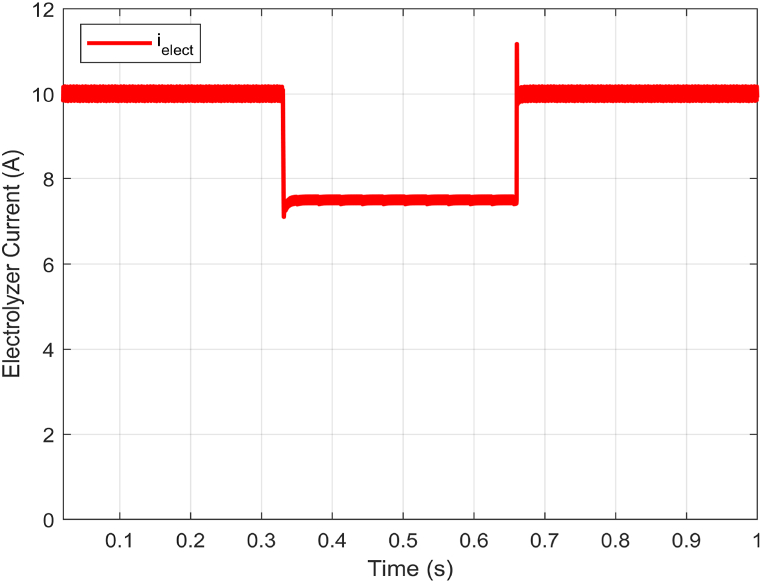
Electrolyzer current ($i_{elect}$) response with electrolyzer reference voltage variations.

Fig. 8 illustrates the electrolyzer voltage tracking behavior, while Fig. 9 displays the electrolyzer current tracking behavior. Both figures showcase the performance of the proposed controller during reference variations. Specifically, an abrupt change from the nominal value of 8V to 6V occurs in the reference voltage, which then compensates. As seen in Fig. 8, the ABTSMC-PINN controller can smoothly track the new reference trajectory of 6V in just about 34ms, even amidst such uncertainty. The excellent compensation precision of the PINN allows for faster rejection of reference voltage variations, resulting in better voltage tracking performance.

### 4.3 Input DC Bus Voltage Fluctuations

**Fig. 10:**
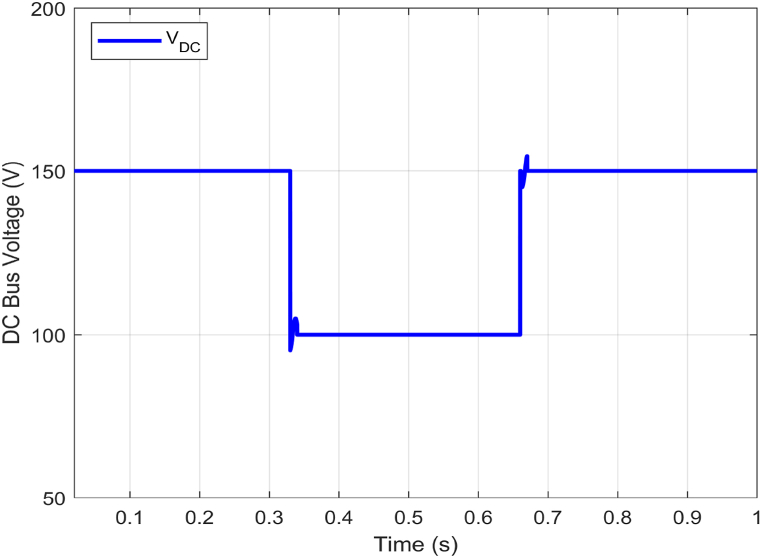
Dc Bus voltage ($V_{DC-Bus}$) fluctuations.

**Fig. 11:**
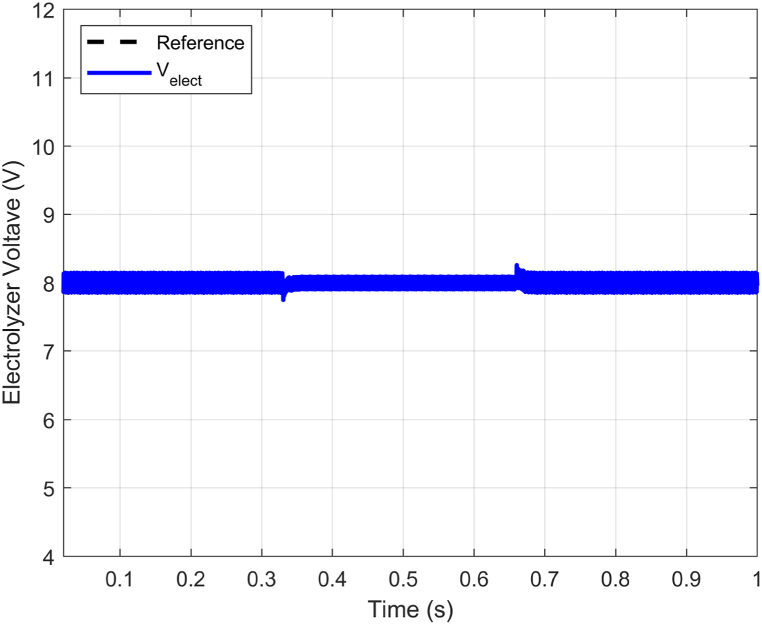
Electrolyzer voltage ($V_{elect}$) response with DC Bus voltage fluctuations.

**Fig. 12:**
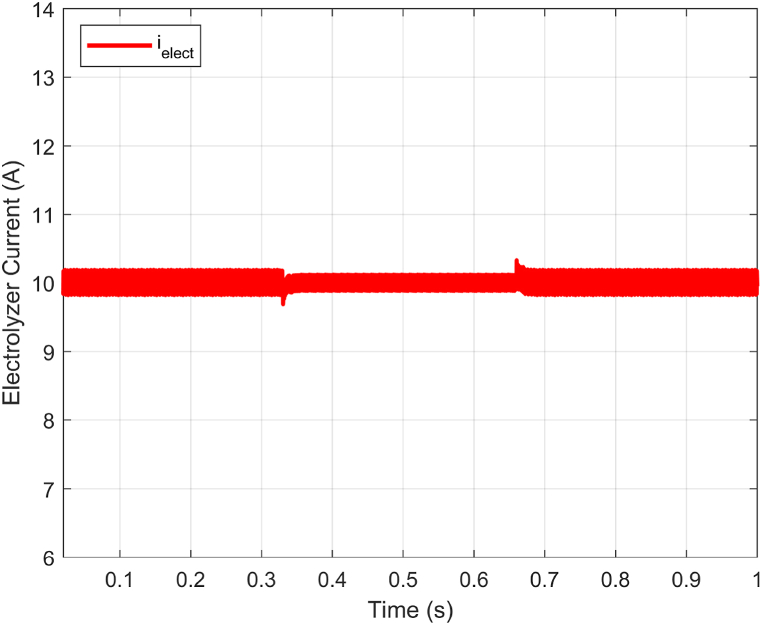
Electrolyzer current ($i_{elect}$) response with DC Bus voltage fluctuations.

The proposed ABTSMC-PINN controller's ability to stabilize the electrolyzer voltage was put to the test under dynamic operation conditions. To simulate the unpredictable behavior of the DC microgrid components to show the effectiveness in case of RESs output variations due to irradiance and wind speed uncertainty, this scenario illustrates the operational performance of the proposed controller under time-varying disturbance. In Fig. 10, an input voltage variation from 150V to 100V with some fluctuations during transients is introduced to approximate the DC bus voltage changes, which may compromise electrolyzer voltage stability. The variation is introduced within the proposed $V_{DC-Bus}$ range (100−200V). Notably, electrolyzer current and voltage ripples remain minimal during steady-state operation (see Fig. 11 and Fig. 12).

Furthermore, the proposed PEM electrolyzer model operates in a constant voltage mode, with the electrolyzer voltage $V_{elect}$ maintained at the rated level of 8V and a constant electrolyzer current $i_{elect}$ of 10A. Additionally, the $i_{elect}$ has very minimal ripples (see Fig. 12). In summary, $V_{elect}$ remains stable even during fluctuations, ensuring the proposed approach's significant benefits and guaranteeing the power electronics components' reliability (see Fig. 11).

### 4.4 Comparative analysis

**Fig. 13:**
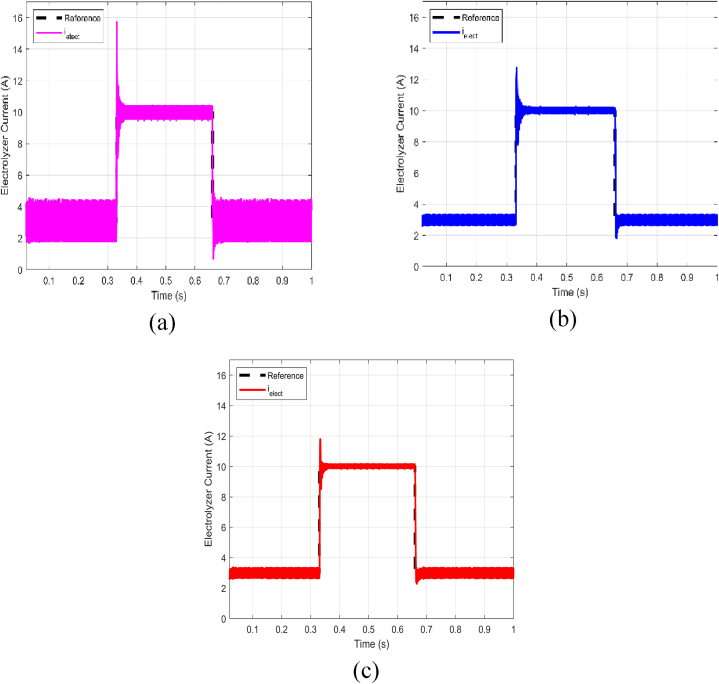
Comparison results: (a) Ismc. (b) ABTSMC-DHLRNN. (c) Proposed ABTSMC-PINN

In order to conduct a thorough comparison between the proposed ABTSMC-PINN controller and recently published controllers, we carefully tuned the parameters of two other controllers: the improved sliding mode-based controller (ISMC) [51] and an ABTSMC controller based on a double hidden layer recurrent neural network (DHLRNN) [52]. The goal was to achieve optimal performance and guarantee accurate and thorough comparisons between the control laws. The control strategies were then experimentally tested under conditions of 100V DC bus voltage to replicate different power sources for the proposed electrolyzer system. Additionally, in order to evaluate the controllers' performance for larger current steps, we increased the electrolyzer current, $i_{elect}$, from 3A to 10A. Fig. 13 shows the outcomes of these tests.

As evident in Fig. 13, the ABTSMC-PINN controller surpasses its counterparts in terms of performance. It facilitates reaching the desired current value faster during both transient phases while ensuring the system's stability. Table 4 presents the performance metrics of the three controllers under comparison. Under varying operational conditions, the proposed ABTSMC-PINN controller demonstrates rapid response, achieving a settling time of just 0.025 s. Moreover, it exhibits minimal overshoot, approximately 17.5%, and maintains stability with minimal fluctuations.

**Table 4:** Performance evaluation of the proposed ABTSMC-PINN controller in comparison to the ISMC and ABTSMC-DHLRNN controllers.

Characteristics	ISMC	ABTSMC-DHLRNN	ABTSMC-PINN
Maximum Overshoot (%)	58	26.5	17.5
Settling Time (s)	0.043	0.038	0.025
Fluctuations (A)	Very high	Low	Low

**Fig. 14:**
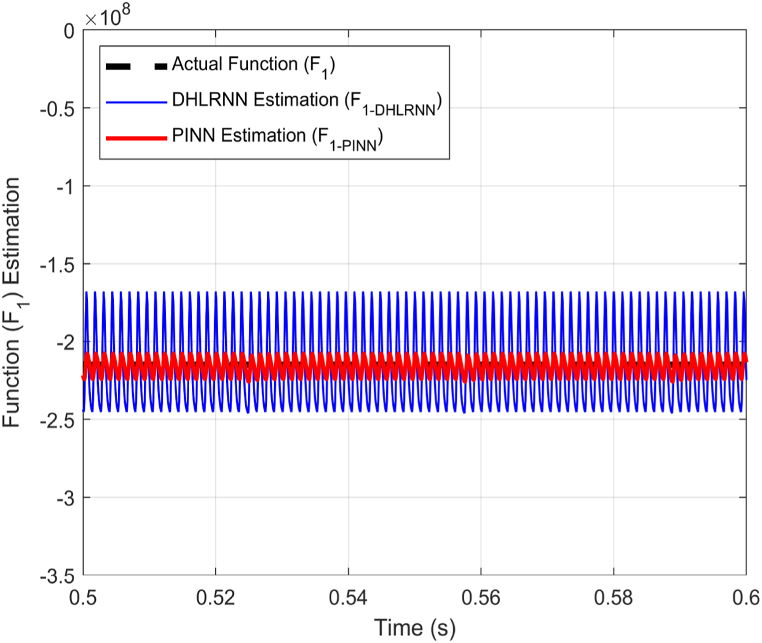
Estimation of the uncertain function ($F_{1}$).

This paper proposes utilizing the PINN for estimating the nonlinear functions ($F_{1}\;\&\;F_{2}$), To enrich the HIL experimental results, a comparison between the actual function $F_{1}$, its DHLRNN prediction, and its PINN prediction is presented in Fig. 14. The results indicate that the PINN offers high estimation precision, making it an effective solution for compensating for system uncertainty by incorporating the estimated value into the control law.

In conclusion, the proposed system represents a significant advancement in the field of PEM electrolyzer operation. Through the design of advanced nonlinear controller, it has the potential to make a meaningful impact and provides a reliable solution to enhance the performance of hydrogen production systems.

While the proposed ABTSMC-PINN controller shows promise, there are some limitations to consider. For optimal regulation and closed-loop system stability, all parameters of the PINN must be tuned offline. However, this approach can be computationally intensive. Additionally, the PINN structure is currently configured through trial-and-error, which can also lead to efficiency challenges if a large number of neurons is selected. Moving forward, it is imperative for future studies to delve into advanced control strategies aimed at diminishing computational loads and enhancing overall algorithmic efficiency.

## 5 Conclusion

A cutting-edge approach has been proposed to enhance the stability of DC power conversion by using PINN in conjunction with a composite ABTSMC approach for voltage tracking control of a buck power converter connected with a PEM electrolyzer. This approach utilizes PINN for nonlinear function estimation, which can effectively eliminate the negative impact of system uncertainty by incorporating the above estimates into the controller. Additionally, it can reduce the sliding mode's switching term, which helps alleviate the chattering phenomenon. The TSMC control, which combines the advantages of backstepping approach and terminal sliding mode design, reduces the complexity of control design and attains the property of finite-time convergence for the tracking error. The superiority of the proposed approach has been demonstrated under multiple operating conditions through HIL implementation results. By prolonging the life and improving the energy efficiency of the PEM electrolyzer, the proposed design significantly increased the productivity of the hydrogen production system. This approach holds great potential for higher power electrolyzers, promising enhanced productivity and efficiency and offering exciting prospects for practical application in hydrogen production systems. In the future, it is crucial for further research to focus on advanced control systems that aim to reduce computing burdens and improve overall algorithmic efficiency.

## Data availability statement

Data will be made available on request.

## CRediT authorship contribution statement

**Abdullah Baraean:** Writing – original draft, Visualization, Software, Methodology, Formal analysis, Data curation, Conceptualization. **Mahmoud Kassas:** Writing – review & editing, Validation, Supervision, Resources, Project administration, Methodology, Investigation, Funding acquisition, Conceptualization. **Md Shafiul Alam:** Writing – review & editing, Visualization, Resources, Methodology, Formal analysis, Conceptualization. **Mohamed A. Abido:** Writing – review & editing, Validation, Supervision, Resources, Project administration, Methodology, Investigation, Funding acquisition, Formal analysis, Conceptualization.

## Declaration of competing interest

The authors declare that they have no known competing financial interests or personal relationships that could have appeared to influence the work reported in this paper.

## Figures and Tables

**Table 5:** Controller Parameter Values

Controllers	Parameters & Values
ISMC	$K_{i}=\lambda_{i}=$ 1256, $K_{V}=\lambda_{V}=$ 125
ABTSMC-DHLRNN	$c=2*10^{5}\text{,}$k=4000, h=2000, β=1200, η=0.1,T=0.01
Proposed ABTSMC-PINN	$K_{1}=10\text{,}$$K_{2}=50$, $K_{3}=30$, $K_{4}=15$, K=1.4

## Appendix

To compare the proposed ABTSMC-PINN with the other controllers, the sliding surfaces of the ISMC and ABTSMC-DHLRNN are expressed as follows:

First, for the ISMC, the output inductor current ($i_{o}$), can be employed in an improved sliding equation as follows:

$$S_{\text{ISMC}}=I_{\text{oref}}-i_{o}+K_{i}\int \left(I_{\text{oref}}-i_{o}\right)dt$$

With a reaching law defined as:

$${\dot{S}}_{\text{ISMC}}=-\lambda_{i}\;S_{\text{ISMC}}$$

The improved sliding equation to balance the electrolyzer voltage ($V_{elect}$) control loop can be applied in the same form as for the inductor current control loop.

Second, for the ABTSMC-DHLRNN. To guarantee the finite-time convergence of tracking errors, the terminal function P(t) is introduced. Thus, the backstepping variables $z_{1}$ and $z_{2}$ are redefined as

$$z_{1}=X_{1}-V_{\text{ref}}-P=e-p$$

$$z_{2}=X_{2}+{cz}_{1}-{\dot{V}}_{\text{ref}}-\dot{p}$$

With regard to the definition of the terminal function P(t), for the sake of ensuring global robustness of the system, let e(0)=P(0), $\dot{e}\left(0\right)=\dot{p}\left(0\right)$. Moreover, in order to ensure that the tracking error can obtain finite-time convergence, when t≥T, the condition of P(t)=0, $\dot{p}\left(t\right)=0$, $\ddot{p}\left(t\right)=0$ should hold. Thus, the terminal function P(t), can be constructed as follows:

$$z_{1}=\{X_{1}-V_{\text{ref}}-P=e-p$$

$$P\left(t\right)=\left\{ \begin{array}{c} e\left(0\right)+\dot{e}\left(0\right)t+\frac{1}{2}\ddot{e}\left(t\right)t^{2}-\left(\frac{a_{00}}{T^{3}}e\left(0\right)+\frac{a_{01}}{T^{2}}\dot{e}\left(0\right)+\frac{a_{02}}{T}\ddot{e}\left(t\right)\right)t^{3}+\left(\frac{a_{10}}{T^{4}}e\left(0\right)+\frac{a_{11}}{T^{3}}\dot{e}\left(0\right)+\frac{a_{12}}{T^{2}}\ddot{e}\left(t\right)\right)t^{4}-\left(\frac{a_{20}}{T^{5}}e\left(0\right)+\frac{a_{21}}{T^{4}}\dot{e}\left(0\right)+\frac{a_{22}}{T^{3}}\ddot{e}\left(t\right)\right)t^{5},0\leq t\leq T\\ 0,t>T \end{array}\right.$$

where $a_{ij}$ (i,j=0,1,2) are the equation coefficients.

Then, the sliding surface is defined as follows:

$$S_{\text{ABTSMC}-\text{DHLRNN}}=Kz_{1}+z_{2}$$

In addition, to ensure a fair comparison between the three control laws, they implemented on identical test conditions, and their parameters were carefully tuned to achieve optimal performance, as listed in Table 5.
